# Effect of Temperature on the Complex Modulus of Mg-Based Unidirectionally Aligned Carbon Fiber Composites

**DOI:** 10.3390/ma15217812

**Published:** 2022-11-05

**Authors:** Stanislav Kúdela, Juraj Koráb, Pavol Štefánik

**Affiliations:** Institute of Materials and Machine Mechanics, Slovak Academy of Sciences, Dúbravská cesta 9, 845 13 Bratislava, Slovakia

**Keywords:** magnesium composites, carbon fibers, unidirectional composites, dynamic mechanical analysis, damping capacity

## Abstract

Composite materials based on magnesium–lithium (MgLi) and magnesium–yttrium (MgY) matrices reinforced with unidirectional carbon fibers were prepared using the gas pressure infiltration method. Two types of carbon fibers were used, high-strength PAN-based T300 fibers and high-modulus pitch-based Granoc fibers. The PAN-based carbon fibers have an internal turbostratic structure composed of crystallites. The pitch-based carbon fibers have a longitudinally aligned graphite crystal structure. The internal carbon fiber structure is crucial in the context of the interfacial reaction with the alloying element. There are various mechanisms of bonding to carbon fibers in the case of magnesium–lithium and magnesium–yttrium alloys. This paper presents the use of the DMA method for the characterization of the role of alloying elements in the quality of interfacial bonding and the influence on the complex modulus at increasingly elevated temperatures (50–250 °C). The complex modulus values of the composites with T300 fibers were in the range of 118–136 GPa. The complex modulus values of the composites with Granoc fibers were in the range of 198–236 GPa. The damping capacity of magnesium-based unidirectionally aligned carbon fiber composites is related to the quality of the interfacial bonding.

## 1. Introduction

The automotive and aerospace industries desire low-gravity structural materials. Magnesium alloys are widely applied in the automotive industry, including in transfer cases, radiator supports, instrument panel beams, and steering components [1,2,3,4]. Magnesium alloys have the lowest specific gravity of all structural alloy metals. Magnesium alloys have a minimal density and are used in mass saving applications to replace other metals. The strength and elastic modulus of magnesium alloys are low. The properties of magnesium alloys can be significantly enhanced via the addition of particles, fibers, nanofibers, and nanotubes to form metal matrix composites (MMC) [5,6,7,8,9,10]. The Young’s modulus of magnesium alloys can be increased via the particles, whiskers, and fibers. The chemical reaction between the matrix and the reinforcing phase improves the interfacial bonding strength [5]. The tensile properties and toughness of the magnesium alloys can be improved via the addition of small amounts of CNTs and SiC nanoparticles [6]. Upadhyay et al. highlighted the poor wettability between CNTs and the magnesium matrix. The interfacial bonding can be strengthened by improving the wettability by adding Cu, Ni, and Cr as the coatings of the CNTs [7]. Guan et al. outlined the three main drawbacks of magnesium-based nanocomposites, namely the agglomeration of the nanoreinforcement, poor interfacial bonding, and poor corrosion resistance [8]. Dey et al. stated that the addition of fibers in the magnesium matrix improves the tensile strength but reduces the ductility [9]. Trojanová et al. studied the thermally activated processes during the deformation of MgLi composites [10]. MgLi alloys are the lightest alloys, with a density range of 1.25–1.65 g·cm^−3^. They have good levels of specific strength and rigidity. This makes them interesting for structural applications, especially in the aerospace and automotive industries. These alloys also have good damping properties and provide good shielding for electromagnetic radiation. The good properties of MgLi alloys can be improved by adding a reinforcing phase (particles, short fibers, or long fibers). The active chemical nature of MgLi alloys presents certain difficulties in the preparation of composites [11].

Carbon fibers are suitable for the reinforcement of magnesium alloys. In general, we can say that there are two basic groups of carbon fibers. PAN-based carbon fibers have an internal turbostratic structure comprised of crystallites. PAN-based carbon fibers have tensile modulus values in the range of 230 to 588 GPa. Pitch-based carbon fibers have a longitudinally aligned graphite crystal structure and have tensile modulus values in the range of 840 to 965 GPa [12].

In Mg/CF composites, the key role is played by the interface between the matrix and the reinforcing phase, which affects the properties of the composite. The key features of the interface are the chemical reaction and bond strength. The nature of the fiber/matrix interface is determined by the matrix alloy, the type of carbon fiber, and its internal structure [13,14].

Due to the high affinity between Li and CF, it is necessary to form a barrier layer on the surfaces of the fibers, usually of pyroC or SiC, in order to prevent damage to the carbon fibers [15]. However, this process is time-consuming and expensive. By adhering to certain technological parameters, it is possible to achieve bending strengths of up to 1200 MPa [16].

Unidirectionally aligned composites are suitable for flexural loading applications. Such composites can be used for thin-walled applications as device cases or tubes. Thin-walled device cases are usually locally thermally and mechanically loaded. It is beneficial to know the limits for everyday use.

A dynamic mechanical analysis is a non-destructive method that allows the testing of prismatic samples in the temperature range of −150–600 °C and the frequency range of 0.01–200 Hz. This method allows one to follow the development of the interfacial bonding quality in relation to the temperature. This method is usually exploited when testing viscoelastic materials [17,18,19]. There have been several studies focusing on the dynamic mechanical properties of particle-reinforced metal matrix composites [20,21,22] and ceramic composite materials. There is lack of knowledge about the dynamic mechanical properties at elevated temperatures in metal matrix fibrous composites.

Here, we describe the temperature development of the dynamic Young’s modulus (complex modulus) values of unidirectionally aligned carbon-fiber-reinforced composites. There are two types of carbon fibers (PAN-based T300 fibers and pitch-based Granoc fibers). Carbon fibers have different internal structures, which influences the interfacial bonding. Lithium and yttrium as alloying elements in magnesium have different chemical mechanisms of interfacial bonding. There are four individual species of interfacial bonding. The influence of the interfacial bonding on the development of the complex modulus at elevated temperatures will be presented here. Composites prepared using the gas pressure infiltration method will be inspected using a dynamic mechanical analysis (DMA). The microstructures of the composites will be presented. The interfacial bonding quality and its influence on the complex modulus at elevated temperatures will be discussed.

The results obtained here might contribute to the knowledge on the influence of interfacial bonding on the dynamic mechanical properties at elevated temperatures.

## 2. Materials and Methods

### 2.1. Magnesium Alloy Preparation

The Mg2 wt.% Li (Mg2Li) alloy was prepared via the re-melting of pure magnesium (99.9%, LMT Metalurgie, Laakirchen, Austria and lithium (99.0% Merck spol. s.r.o., Bratislava, Slovakia) in a mild steel crucible with stirring under an argon (purity 99.999%, Messer Tatragas, Bratislava, Slovakia) atmosphere (1 MPa) in the autoclave after the previous evacuation (10 Pa). The magnesium–yttrium alloy (Mg1.8 wt.%Y, IMSAS, Bratislava, Slovakia) was prepared in the same way using pure magnesium (99.9%, LMT Metalurgie, Laakirchen, Austria) and yttrium (99.0% Alfa Aesar, Karlsruhe, Geramany). The alloy markings Mg1.8 wt.%Y and Mg2 wt.% Li indicate the alloy category. The determination of the alloy composition via an atomic emission spectral analysis is regularly performed. In the case of Mg1.8Y, the composition varied in the range of 1.73–1.89 wt.% Y, and in the case Mg2Li, the composition varied in the range of 1.91–2.06 wt.%. An EDX analysis of the matrix was performed [23]. The oxygen content range in the MgLi matrix was 0.51–0.62 wt.%. It has to be noted that the surface analysis was performed after exposure to air. We consider the oxygen content in the magnesium alloy matrix to be low.

### 2.2. Carbon Fibers (T300, Granoc)

**Figure 1:**
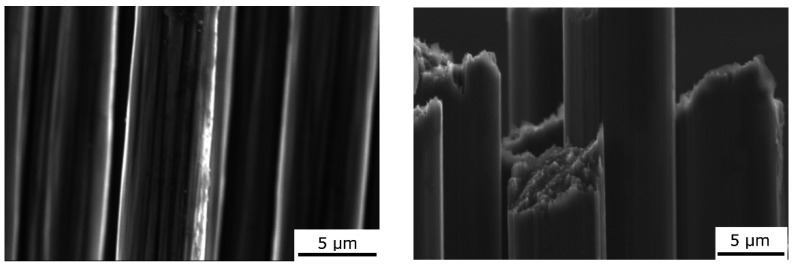
SEM images of the carbon fibers. PAN-based T300 (**left**) and pitch-based Granoc (**right**) fibers.

Two types of carbon fibers were used to prepare unidirectional metal matrix composites. Torayca T300 (Toray, Tokyo, Japan) PAN-based carbon fibers (Table 1) [24] and XN90-60S pitch-based Granoc fibers (Nippon Graphite, Otsu-shi, Shiga-ken, Japan) (Figure 1) were used (Table 1) [25]. The structure of the T300 PAN-based fibers consisted of turbostratic graphite with disordered small graphene-like blocks. In contrast, the structure of the pitch-based Granoc fibers consisted of large graphene layers with an aligned orientation [5,26].

A fibrous preform (15 × 15 × 70 mm^3^) made of perforated steel sheets with 50 vol. % unidirectionally arranged carbon fibers was prepared for the infiltration process.

### 2.3. Gas Pressure Infiltration Method

**Figure 2:**
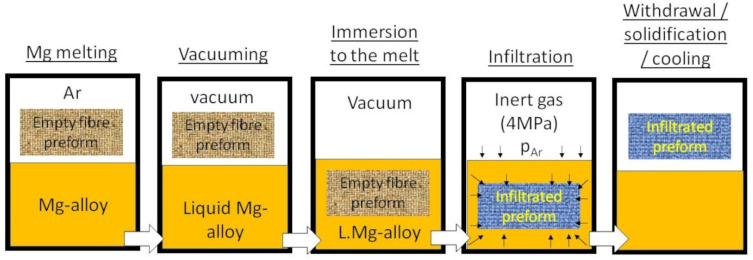
Schematic image of the gas pressure infiltration process.

**Figure 3:**
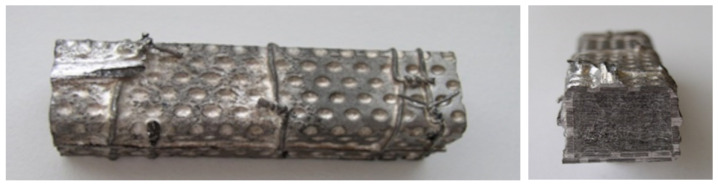
The infiltrated fibrous preform (**left**) and cross-section of the infiltrated fibrous preform (**right**).

The Mg-based carbon fiber composites were fabricated using the gas pressure infiltration method. The method is based on the immersion of an evacuated and preheated fibrous preform into the molten metal. After the immersion, the pressure of an inert gas (argon) is applied on the surface of the melt. The molten metal penetrates into the inter-fiber gaps. The molten metal is then in contact with the fibers for a certain amount of time. Subsequently, the sample is withdrawn and the molten metal spontaneously solidifies (Figure 2) [23,27]. The fibrous preform (15 × 15 × 70 mm^3^) contained unidirectionally arranged carbon fibers (50 vol.%). Figure 3 demonstrates the infiltrated preform.

The interfacial bond quality strongly depends on the infiltration parameters and alloying elements [16,23]. We prepared two types of interfacial bond using two alloying elements (Y, Li) and certain infiltration parameters. The MgY/CF composites were prepared at 900 °C and 4 MPa for 300 s, while the MgLi/CF composites were prepared at 750 °C and 4 MPa for 30 s.

Magnesium and lithium strongly evaporate during the melting process. MgLi alloys must be prepared with a higher pressure of inert gas. It is possible to achieve this in a closed vessel. The gas pressure infiltration technology allows the re-melting of the MgLi alloy and subsequent penetration into the inter-fiber gaps. At the beginning of the whole preparation process for the composite material, it is necessary to evacuate the volume of the vessel to free-up the inter-fiber gaps and to avoid fast oxidation.

### 2.4. Dynamic Mechanical Testing

A dynamic mechanical analysis is a thermal mechanical analysis technique that measures the properties of materials as they deform under periodic stress as a function of temperature (or time). The dynamic mechanical tests were performed in a TA Instruments Q800 dynamic mechanical analyzer (TA Instruments, New Castle, DE, USA). The three-point bending mode was applied in the temperature range of 50–250 °C perpendicularly to the fiber direction. The sample dimensions were 2.5 × 4.0 × 55 mm^3^. The cyclic load was adjusted to 3.5 N and the frequency to 1 Hz. A heating rate of 3 °C/min was applied during heating. In the case of pure Mg, the cyclic load was adjusted to 1 N.

### 2.5. SEM and TEM Observations and EDX Analysis

The structures of the Mg/CF, MgY/CF, and MgLi/CF composites were examined using a scanning electron microscope (SEM, Jeol JSM 7600F)(JEOL, Tokyo, Japan). An energy-dispersive X-ray spectrometer (EDX) (JEOL, Tokyo, Japan) operating at 15 kV was used for the elemental analysis. The composite samples were metallographically polished using water-free isopropyl alcohol to a mirror-like finish without any etching. As the structures of the MgLi/CF and MgY/CF composites are sensitive to atmospheric humidity, they were exposed to air for a limited time (≤1 h) before being inspected via SEM and EDX. The TEM observations were performed with an FEI Themis ETEM–FEG scanning transmission electron microscope (Thermo Fisher Scientific, Waltham, MA, USA) 

## 3. Results and Discussion

### 3.1. Composite Structure

Reaction bonds improve the stress transfer from the matrix to the fiber. Such bonds are desired in metal matrix composite systems. Our previous studies [16,23] aimed to influence lithium and yttrium as alloying elements to the interfacial bond in the MgLi/CF and MgY/CF systems. Li penetrates the bulk fibers, producing lithium carbide Li_2_C_2_ within the fibers. We observed a massive segregation of Y around the carbon fibers [16]. The interfacial product was found around the carbon fibers and the mechanical properties of MgY/CF composites was significantly improved compared with the Mg/CF composite [28,29]. 

#### 3.1.1. Structure of Mg1.8Y/CF Composites

Zhang et al. [28] reported that the addition of small amounts of yttrium to the Mg matrix (1 wt.%) significantly improved the mechanical properties of the MgY/CF composite compared with the Mg/CF composite. Zhang et al. reported on the segregation of alloy element Y in the interfacial fiber/matrix area. They identified nanoscale interfacial layers of Mg_2_Y. The authors did not mention the presence of YC_2_ or Y_2_C in the case of the higher Y content (3.2–8.5 wt.%) in Mg alloy [28,29]. 

Zhang et al. [28,29] observed that Y was prone to segregate near the fiber/matrix interface and formed a nanoscale interfacial layer identified as Mg2Y. A significant improvement of the mechanical properties was confirmed.

**Figure 4:**
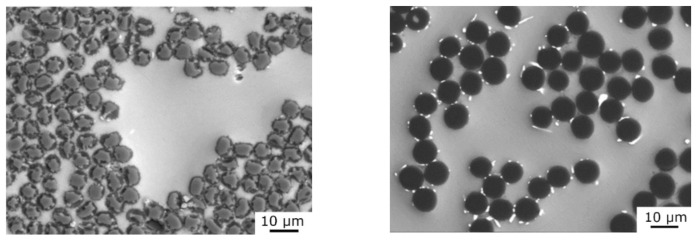
Typical microstructure of the Mg1.8Y/T300 (**left**) and Mg1.8Y/Granoc (**right**) composites.

Figure 4 demonstrates the typical microstructures of Mg1.8Y/T300 and Mg1.8Y/Granoc composites. In the microstructure of Mg1.8Y/T300, an interfacial area with a different morphology compared to the matrix is visible, which was later identified as containing Y-enriched zones. In the microstructure of the Mg1.8Y/Granoc composite, we observed bright particles later identified as Y-enriched formations.

**Figure 5:**
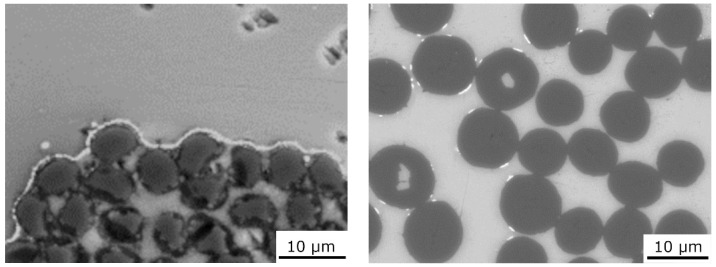
Microstructure of the Mg1.8Y/T300 (**left**) and Mg1.8Y/Granoc (**right**) composites.

The bright areas in Figure 5 in the microstructures of the composites demonstrate the occurrence of a new phase in the interfacial areas in the composites with both T300 and Granoc fibers. 

**Figure 6:**
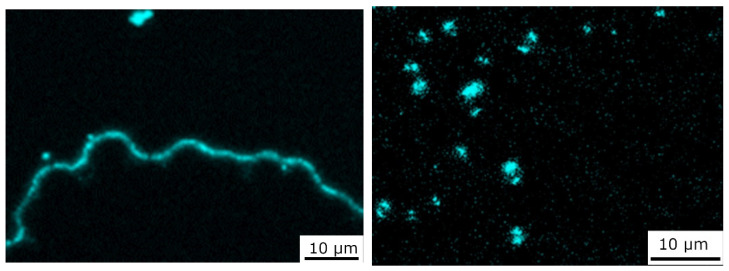
EDX yttrium distributions of Mg1.8Y/T300 (**left**) and Mg1.8Y/Granoc (**right**) composites.

Figure 6 demonstrates the segregation of the alloying element Y using the EDX distribution maps. The occurrence of the segregated yttrium in the interfacial area of the fiber/matrix corresponds with the occurrence of the new bright phase observed in the SEM microstructures in both composites.

**Figure 7:**
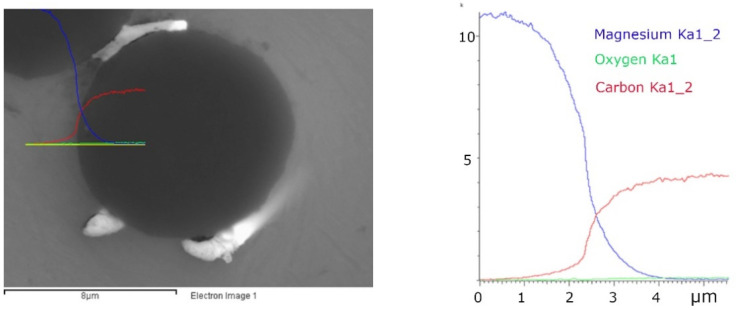
A line scan analysis of the fiber/matrix interface of the Mg1.8Y/Granoc composite without the reaction product. Analyzed interfacial area (**left**) and the signal intensity diagram of analyzed interfacial area (**right**). Colored lines are related to analyzed elements C (red), O (green), Mg (blue), signal intensity is measured in kilocounts per second (kcps) plotted on the y-axis.

**Figure 8:**
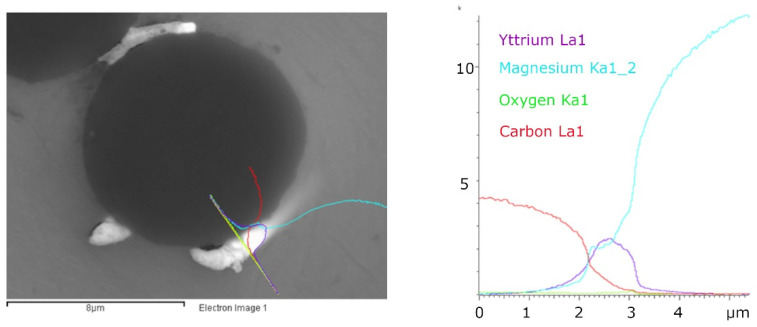
A line scan analysis of the fiber/matrix interface of the Mg1.8Y/Granoc composite with the reaction product. Analyzed interfacial area (**left**) and the signal intensity diagram of analyzed interfacial area (**right**). Colored lines are related to analyzed elements C (red), O (green), Mg (cyan), Y (violet), signal intensity is measured in kilocounts per second (kcps) plotted on the y-axis.

The line scan analysis through the interface (Figure 7) shows that the carbide formation in the interface cannot be detected using this method (if any layer had formed, it was very thin). If the line analysis is performed through a particle that is close to the fiber (Figure 8), the course of the curves of the individual elements will be significantly different. The concentrations of elements Y and C indicate that the formed particle is yttrium carbide.

**Figure 9:**
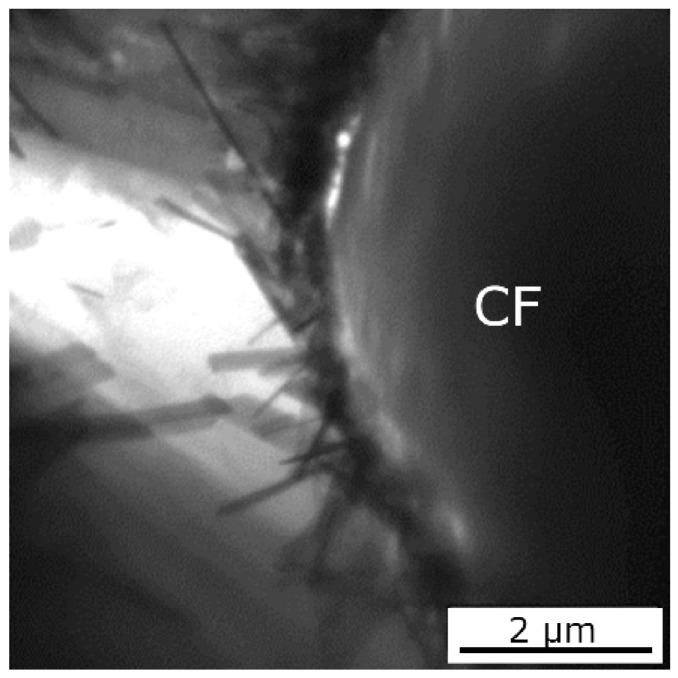
TEM image of the MgY/Granoc interface.

The TEM observation of the fiber/matrix interface of the MgY/Granoc composite demonstrates the occurrence of needle-like phases (Figure 9). The needle-like reaction products in the interface contain yttrium, but the composition and structure of these products cannot be exactly identified. These phases are probably formed from yttrium carbides. The air humidity reacts with the yttrium carbide and decomposes it [16].

#### 3.1.2. Structure of the Mg2Li/CF Composites

**Figure 10:**
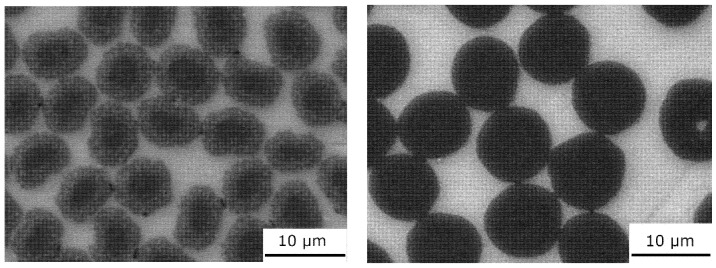
Microstructures of Mg2Li/T300 (**left**) and Mg2Li/Granoc (**right**) composites.

**Figure 11:**
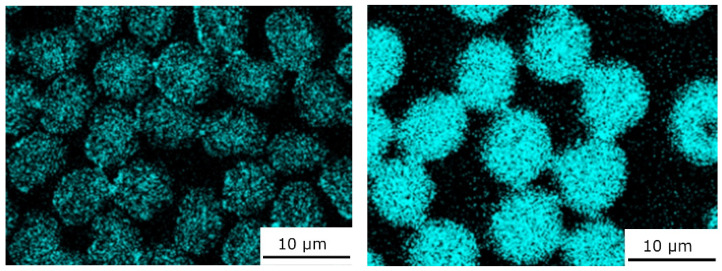
EDX oxygen distribution of Mg2Li/T300 (**left**) and Mg2Li/Granoc (**right**) composites.

Figure 10 shows microstructure Mg2Li/CF composites. Previous investigations [16,23] demonstrated that the EDX oxygen distribution on the fiber cross-section can be used as a rough indicator of the presence of Li_2_C_2_ in carbon fibers. Lithium atoms penetrate inside carbon fibers and rapidly create Li_2_C_2_. Lithium carbide is formed inside fibers, and such reactive interfacial bonding does not appear in any interfacial products. Figure 11 shows strong and uniform Okα signals within the whole-fiber cross-section. 

**Figure 12:**
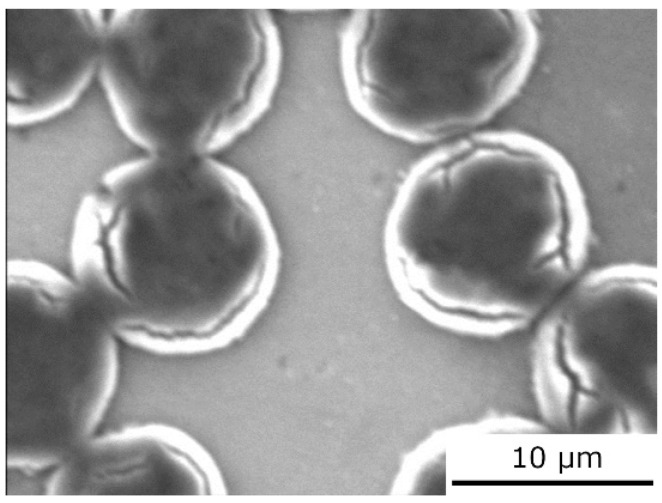
SEM image of the Mg4Li/Granoc composite.

Previous results [16,23] showed that the infiltration parameters (temperature and time) and amount of alloying element influence the quality of the interfacial bonding and concentration of lithium inside the carbon fibers. The large amount of lithium atoms and subsequent creation of Li_2_C_2_ can damage the carbon fibers (Figure 12). The cracking of carbon fibers as a consequence of extensive Li_2_C_2_ creation negatively influences the mechanical properties of the individual carbon fibers. 

Previous studies conducted with Li alloy Mg matrices (8–12 wt.% Li) revealed the destruction of CF due to the excessive formation of lithium carbide Li_2_C_2_ [15].

#### 3.1.3. Composite Structure after the DMA Measurements

**Figure 13:**
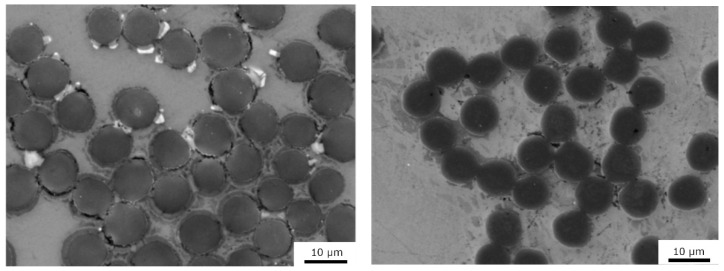
SEM images of Mg1.8Y/Granoc (**left**) and Mg2Li/Granoc (**right**) composites after thermal exposition in the DMA apparatus.

The temperature has a dominant influence on the microstructure of the composites due to thermal expansion coefficient mismatch of the matrix and the fibers. During heating and cooling, the shear stress of the interfacial bonding occurs. There is a partial delamination of the matrix from the fibers (Figure 13), which is caused by the lower efficiency of the stress transfer between the fiber and the matrix (0.44 and 0.49). The DMA measurements at elevated temperatures did not change the microstructures of the Mg1.8Y/T300 and Mg2Li/T300 composites. In these composites, the efficiency rates of the stress transfer between the fiber and matrix are significantly higher (1.03 and 0.89).

### 3.2. Dynamic Mechanical Properties

The mechanical behavior of the unidirectional metal matrix composite does not only depend on the mechanical properties of the individual constituents but more essentially on the strength of the fiber/matrix interface.

In Mg-based CF composites, the key role is played by the interface between the matrix and the reinforcing phase, which affects the overall properties of the composite. A key feature of the interface is the chemical reaction and bond strength. The nature of the fiber/matrix interface is determined by the matrix alloy and the type of carbon fiber [16,23].

Young’s modulus of fiber reinforced unidirectionally aligned composites is determined by Young’s modulus of individual constituents (1).

E_c_ = E_f_V_f_ + E_m_V_m_ (1)

Here, E_c_, E_f_, and E_m_ are theYoung’s moduli of the composite, fiber, and matrix, respectively; V_f_ and V_m_ are the volume portions of the fibers and matrix, respectively.

The complex modulus (dynamic Young’s modulus) plays an important role in the context of the application of metal matrix composites in the automotive and aerospace industries. The structural elements are subjected to vibrations at elevated temperatures. Materials with a high complex modulus, low density, and thermal stability are desired for these applications.

The complex modulus (dynamic Young’s modulus) (E) is determined using Equation (2) [21,30,31]:

(2) $$E=\sqrt{E_{storage}^{2}+E_{loss}^{2}}$$

#### 3.2.1. Complex Modulus of Magnesium

The complex modulus of pure magnesium monotonously decreases across the temperature range of 50–250 °C and the interval range of 35–31 GPa. Munitz at al. investigated the storage and loss modulus of pure magnesium in the specimens parallel and perpendicular to the grains [32]. The specimens parallel to the grains had complex moduli in the temperature range of 50–250 °C and the interval range of 43–34 GPa, while the specimens perpendicular to the grains were in the range of 39–33 GPa. Calculations according to the ROM were performed with a complex modulus range of 35 GPa. 

It is important to note that the Young’s moduli of Mg and MgLi alloys are identical (45 GPa) [33].

There are no data about the Young’s modulus of the Mg1.8Y alloy. On the basis of the earlier results, we can guess that the Young’s modulus of the Mg1.8Y alloy is also identical to that of Mg.

#### 3.2.2. Complex Moduli of Mg-Based CF Composites

**Figure 14:**
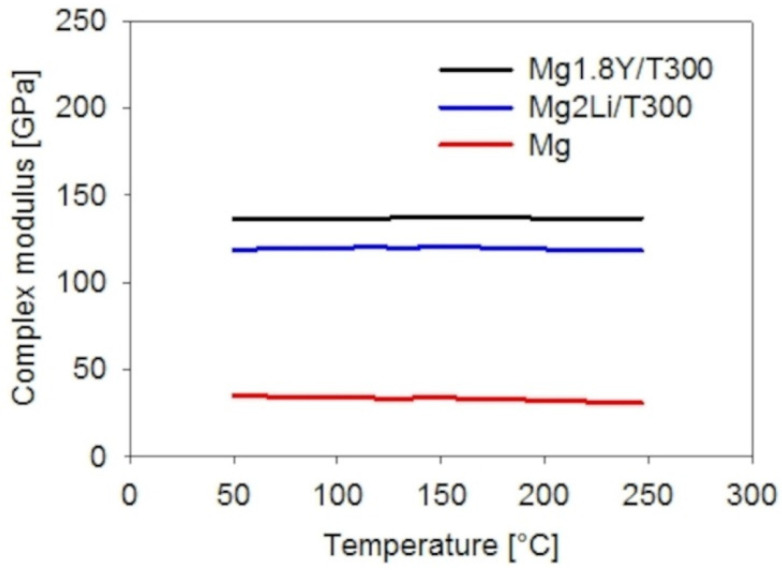
Complex modulus of the composites with the T300 fibers.

The complex moduli of the Mg1.8Y/T300 and Mg2Li/T300 composites in the temperature range of 50–250 °C are shown in Figure 14. Both composites exhibited significant increases in complex modulus compared to pure magnesium. The increase in temperature caused a slight increase in the complex modulus or constant behavior. Both Mg-based T300 composites exhibited highly efficient interfacial bonding as compared to theoretical value determined by the rule of mixtures (ROM) equation (Equation (1)) (Table 2):

The complex modulus values of both presented Mg-based T300 composites are more than 3 times higher compared to pure magnesium and remain more or less constant across the whole investigated temperature range (Figure 14). Both alloying elements Y and Li improved the interfacial bonding to the T300 fibers.

**Figure 15:**
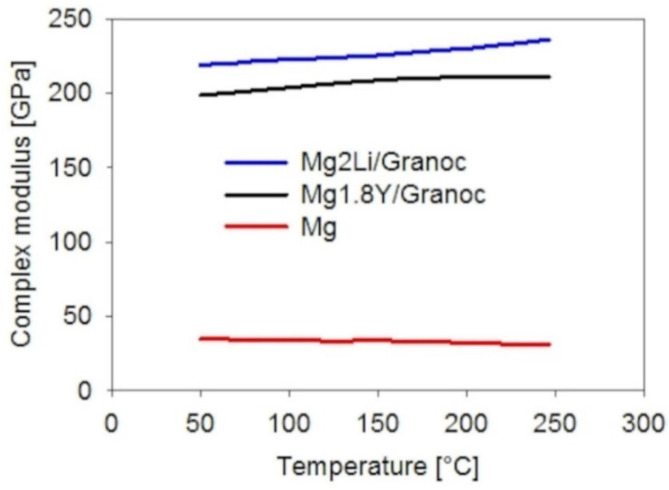
Complex modulus values of the composites with Granoc fibers.

The complex modulus values of composites Mg1.8Y/Granoc and Mg2Li/Granoc across the temperature range of 50–250 °C are shown in Figure 15. Both composites exhibited significant increases in complex modulus values compared to pure magnesium. Both alloying elements Y and Li showed improved interfacial bonding to the Granoc fibers. 

The increase in temperature caused a slight increase in the complex modulus. Both Mg-based Granoc composites exhibited poor efficiency of the interfacial bonding compared to the theoretical value determined by rule of mixtures (ROM) equation (Equation (1)) (Table 2).

The complex modulus results are roughly comparable with the Young’s modulus data from the bending tests [16,23].

#### 3.2.3. Damping capacity of the Mg-Based CF composites

There are several quantities used to characterize the damping capacity. In this work, the damping capacity was evaluated in terms of the tan delta [20].

**Figure 16:**
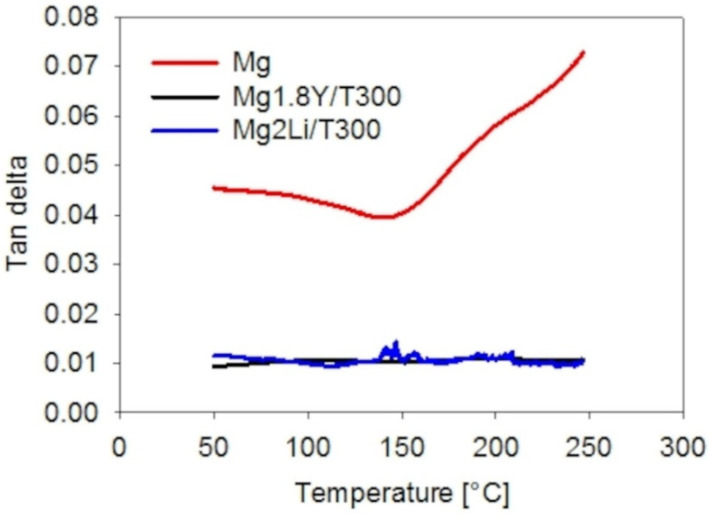
Damping capacity values of the composites with T300 fibers.

The damping capacities of the Mg-based T300 composites were much lower than pure Mg. Good interfacial bonding corresponded to poor damping capacity for the Mg-based T300 composites (Figure 14 and Figure 16). Composite Mg2Li/T300 exhibited an increase in damping capacity from 0.01 to 0.014 across the interval range of 135–150 °C. This effect was connected with slight increases and decreases in the complex modulus in the interval range of 120–120.6 GPa. The height of the peak was 40% of the average damping capacity value (0.01). Luz et al. showed a 1600% increase in the damping capacity peak and Goertzen at al. published a 4000% increase in the damping capacity peak [17,18]. The described effect can be explained by local balance creation or a local imperfection in the interfacial bonding.

**Figure 17:**
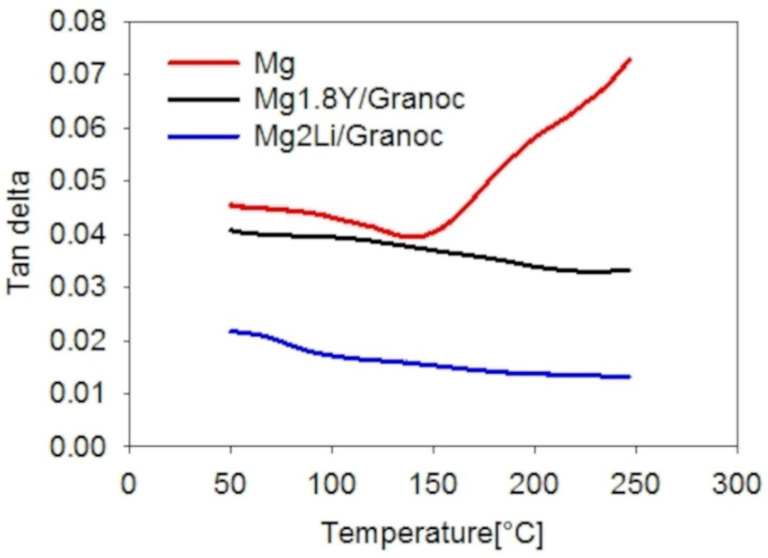
Damping capacity values of the composites with Granoc fibers.

The higher damping capacities of the Mg-based Granoc composites are related to the interfacial bonding quality. The Mg2Li/Granoc composite exhibits a higher complex modulus of 219.1 GPa at 50 °C (Figure 15) (E/E_ROM_ = 0.49) and has a lower damping capacity of 0.02 at 50 °C (Figure 17). The Mg1.8Y/Granoc composite exhibits a lower complex modulus of 198.7 GPa at 50 °C (Figure 15) (E/E_ROM_ = 0.44) and a higher damping capacity of 0.04 at 50 °C (Figure 17).

The damping capacity of the Mg-based composites is higher than the damping capacity of the AZ91D/FAC composites at a test frequency of 1 Hz [20]. The damping capacity of the Mg-based T300 composites remains constant with elevated temperature, while the damping capacity of the Mg-based Granoc composites decreases with elevated temperature. The damping capacity of the AZ91D/FAC composites increased rapidly with the increasing temperature [20]. The dominant factors affecting the complex modulus of the composite are the fibers and the quality of the interfacial bonding. The M1.8Y and Mg2Li matrices influence the overall complex modulus of the composite to a lesser extent. In both the T300 fiber group and the Granoc fiber group, the higher complex modulus confirms the better fiber/matrix bond. Therefore, we relate the better damping properties of the composites to the worse interfacial fiber/matrix bonding.

### 3.3. Interfacial Bond Transfer Efficiency

We can summarize that the load transfer efficiency of the group of Mg-based T300 composites was significantly higher than in the group of Mg-based Granoc composites (Table 2). This can be explained by the ridge-like geometry of the T300 carbon fibers, the higher content of nitrogen (6.96) [34] in the T300 carbon fibers, and the different degrees of ordering of the internal structures of the T300 and Granoc fibers. 

The slight increases in complex modulus values in both types of Granoc fiber composites and the constant complex modulus values in both types of T300 fiber composites over the temperature range introduced here were rather surprising. The complex modulus of the matrix decreased over the temperature range introduced here. The quality of the interfacial bonding was very good in the group of PAN-based T300 fiber composites and sufficient in the group of pitch-based Granoc fiber composites. The rule of mixtures applies to composites [5]. The explanation has to be in the behavior of the carbon fibers. We can make a simple assumption that the dynamic Young’s modulus of the carbon fibers can increase at elevated temperatures compared to the value at room temperature. This effect is more significant in the case of Granoc fibers due to their ordered structure. There is no knowledge about the thermal relation of the dynamic Young’s modulus of the carbon fibers [12,35,36,37]. 

The complex modulus consists of two components, *E_storage_* and *E_loss_*, related to the formula $E=\sqrt{E_{storage}^{2}+E_{loss}^{2}}$. The composites Mg1.8Y/CF and Mg2Li/CF are two orders of magnitude higher in the E_storage_ modulus than the E_loss_ modulus (Table 3). The damping capacity is defined as $\tan\delta =\frac{E_{loss}}{E_{storage}}$. There is straight relation of the complex modulus with the E_storage_ modulus and a direct relation of the damping capacity with the E_loss_ modulus.

## 4. Conclusions

The results of the dynamic mechanical testing of magnesium-based carbon fiber composites in the temperature range of 50–250 °C prepared using the gas pressure infiltration method can be summarized as follows:

(1) Two types of interfacial bonding were presented, reaction bonding with the reaction product in the interfacial area (Mg1.8Y/CF) and reaction bonding without the reaction product in the interfacial area (Mg2Li/CF);
(2) The complex modulus values slightly increased in the temperature range of 50–250 °C in both types of Granoc fiber composites, while in both types of T300 fiber composites the complex modulus values remained constant over the temperature range introduced here;
(3) The highest complex modulus in the group of Granoc fiber composites was for the Mg2Li matrix composites over the temperature range introduced here;
(4) In the group of T300 fiber composites, the highest complex modulus was for the Mg1.8Y matrix composites over the temperature range introduced here;
(5) Both magnesium matrices (Mg1.8Y, Mg2Li) showed better bonding efficiency in the T300 fiber composites compared to the Granoc fiber composites. This can be related to the ridge-like geometry of the T300 carbon fibers, the higher content of nitrogen in the T300 carbon fibers, and the different degrees of ordering in the internal structures of the T300 and Granoc fibers;
(6) D = The damping capacity is related to the quality of the interfacial bonding, whereby lower-quality interfacial bonding results in a higher damping capacity.

## Figures and Tables

**Table 1:** Basic data for the T300 and Granoc carbon fibers [24,25].

Carbon Fibre	Producer	Fiber Type	E-Modulus [GPa]	Tensile Strength [GPa]	Density [g cm^−3^]	Diameter [µm]	CTE [10^−6^ K^−1^]
Torayca T-300	Toray	PAN	230	3.53	1.76	7	−0.41
Granoc XN90-60S	Nippon Graphite Fibre Corporation	PITCH	860	3.50	2.20	10	−1.5

**Table 2:** Dynamic Young’s modulus values of Mg-based T300 and Mg-based Granoc composites.

Sample	E_(ROM)_ Modulus [GPa]	E/E_(ROM)_	E modulus (50 °C) [GPa]	E modulus (150 °C) [GPa]	E modulus (250 °C) [GPa]
Mg1.8Y/T300	132.5	1.03	136.3	137.1	136.7
Mg2Li/T300	132.5	0.89	118.9	120.7	118.1
Mg1.8Y/Granoc	447.5	0.44	198.7	208.6	211.3
Mg2Li/Granoc	447.5	0.49	219.1	225.5	235.9

**Table 3:** Representative values of the storage and loss moduli of Mg-based T300 and Mg-based Granoc composites.

Sample	E_storage_ (50 °C) [GPa]	E_loss_ (50 °C) [GPa]	E_storage_ (150 °C) [GPa]	E_loss_ (150 °C) [GPa]	E_storage_ (250 °C) [GPa]	E_loss_ (250 °C) [GPa]
Mg	34.9	1.6	33.6	1.4	30.9	2.2
Mg1.8Y/T300	136.3	1.3	137.1	1.4	136.6	1.4
Mg2Li/T300	118.9	1.4	120.7	1.3	118.1	1.2
Mg1.8Y/Granoc	198.5	8.1	208.5	7.7	211.2	7.0
Mg2Li/Granoc	219.1	4.7	225.5	3.4	235.9	3.1

## Data Availability

The data are provided within the article.

## References

[1] Liu W., Zhou B., Wu G., Zhang L., Peng X., Cao L. (2019). High temperature mechanical behavior of low-pressure sand-cast Mg–Gd–Y–Zr magnesium alloy. J. Magnes. Alloy.

[2] Kulekci M.K. (2008). Magnesium and its alloys applications in automotive industry. Int. J. Adv. Manuf. Technol..

[3] Tan J., Ramakrishna S. (2021). Applications of Magnesium and Its Alloys: A Review. Appl. Sci..

[4] Luo A.A. (2013). Magnesium casting technology for structural applications. J. Magnes. Alloys.

[5] Shi H., Xu C.H., Hu X., Gan W., Wu K., Wang X. (2022). Improving the Young’s modulus of Mg via alloying and compositing—A short review. J. Magnes. Alloys.

[6] Zhou X., Su D., Wu C., Liu L. (2012). Tensile Mechanical Properties and Strengthening Mechanism of Hybrid Carbon Nanotube and Silicon Carbide Nanoparticle-Reinforced Magnesium Alloy Composites. J. Nanomaterials.

[7] Upadhyay G., Saxena K.K., Sehgal S., Mohammed K.A., Prakash C.H., Dixit S., Buddhi D. (2022). Development of Carbon Nanotube (CNT)-Reinforced Mg Alloys: Fabrication Routes and Mechanical Properties. Metals.

[8] Guan H., Xiao H., Ouyang S., Tang A., Chen X., Tan J., Feng B., She J., Zheng K., Pan F. (2022). A review of the design, processes, and properties of Mg-based composites. Nanotechnol. Rev..

[9] Dey A., Pandey K.M. (2015). Magnesium metal matrix composites—A review. Rev. Adv. Mater. Sci..

[10] Trojanová Z., Drozd Z., Lukáč P., Džugan J. (2021). Studying the Thermally Activated Processes Operating during Deformation of hcp and bcc Mg–Li Metal-Matrix Composites. Metals.

[11] Sun Y., Wang R., Peng C., Feng Y., Yang M. (2019). Recent progress in Mg−Li matrix composites. Trans. Nonferrous Met. Soc. China.

[12] Newcomb B.A. (2016). Processing, structure, and properties of carbon fibers. Compos. Part A Appl. Sci. Manuf..

[13] Körner C., Schäff W., Ottmüller M., Singer R.F. (2000). Carbon long fiber reinforced magnesium alloys. Adv. Eng. Mater..

[14] Ye H.Z., Liu X.Y. (2004). Review of recent studies in magnesium matrix composites. J. Mater. Sci..

[15] Kudela S., Gergely V., Jänsch E., Hofmann A., Baunack S., Oswald S., Wetzig K. (1994). Compatibility between PAN-based carbon fibres and Mg8Li alloy during the pressure infiltration process. J. Mater. Sci..

[16] Kúdela S., Bajana O., Orovčík Ľ., Ranachowski P., Ranachowski Z. (2020). Strengthening in MgLi matrix composites reinforced with unidirectional T300 and Granoc carbon fibres. Kovove Mater..

[17] Goertzen W.K., Kessler M.R. (2007). Dynamic mechanical analysis of carbon/epoxy composites for structural pipeline repair. Composites B.

[18] Luz F.S., Monteiro S.N., Tommasini F.J. (2018). Evaluation of Dynamic Mechanical Properties of PALF and Coir Fiber Reinforcing Epoxy Composites. Mater. Res..

[19] Patra S., Ajayan P.M., Narayanan T.N. (2021). Dynamic mechanical analysis in materials science: The Novice’s Tale. Oxf. Open Mater. Sci..

[20] Yu S.R., Li F.G., Chu H.C., Liu E.Y. (2018). The influence of FAC particle size on the damping capacity and dynamic Young’s modulus of AZ91D/FAC composites at high temperature. Int. J. Mater. Res..

[21] Madeira S., Carvalho O., Carneiro V.H., Soares D., Silva F.S., Miranda G. (2016). Damping capacity and dynamic modulus of hot pressed AlSi composites reinforced with different SiC particle sized. Compos. Part B. Eng..

[22] Acar E., Aydın M. (2021). Damping behavior of Al/SiC functionally graded and metal matrix composites. J. Asian Ceram. Soc..

[23] Kúdela S., Bajana O., Orovčík Ľ., Ranachowski P., Ranachowski Z. (2020). Alloying effect of Li and Y on the strengthening of Mg/T300 composites. Kovove Mater..

[24] (2011). Torayca Data Sheet, Tokio.

[25] Granoc Yarn XN Series, Nippon Graphite Fiber Corporation. www.ngfworld.com.

[26] Qin X., Lu Y., Xiao H., Wen Y., Yu T. (2012). A comparison of the effect of graphitization on microstructures and properties of polyacrylonitrile and mesophase pitch-based carbon fibers. Carbon.

[27] Hufenbach W., Andrich M., Langkamp A.A. (2006). Czulak. Fabrication technology and material characterization of carbon fibre reinforced magnesium. J. Mater. Process. Technol..

[28] Zhang S., Chen G., Pei R., Li D., Wang P., Wu G. (2014). Effect of Y addition on the interfacial microstructures and mechanical properties of Cf/Mg. Mater. Sci. Eng. A Struct. Mater..

[29] Zhang S., Chen G., Pei R., Wang Y., Li D., Wang P., Wu G. (2015). Effect of Y content on interfacial microstructures and mechanical properties of Cf/Mg composite. Mater. Sci. Eng. A Struct. Mater..

[30] Yu W., Li X., Vallet M., Tian L. (2019). High temperature damping behavior and dynamic Young’s modulus of magnesium matrix composite reinforced by Ti2AlC MAX phase particles. Mech. Mater..

[31] Kang C.S., Maeda K., Wang K.J., Wakashima K. (1998). Dynamic Young’s modulus and internal friction in particulate SiC/Al composites. Acta Mater..

[32] Munitz A., Dayan D., Pitchure D., Ricker R., Alan A., Luo T.M.S. (2004). Dynamic mechanical analysis of pure Mg and Mg AZ31 alloy. Magnesium Technology 2004 (The Minerals, Metals & Materials Society).

[33] Chang T.C., Wang J.Y., Chu C.L., Lee S. (2006). Mechanical properties and microstructures of various Mg-Li alloys. Mater. Lett..

[34] https://www.jinjiuyi.net/news/difference-between-t300-t700-and-t800-carbon-fiber.html.

[35] Chand S. (2000). Review Carbon fibers for composites. J. Mater. Sci..

[36] Edie D.D. (1998). The effect of processing on the structure and the properties of carbon fibers. Carbon.

[37] Chung D.D.L. (2002). Review Graphite. J. Mater. Sci..

